# Sexually Transmitted Infections Among Active Component Members of the U.S. Armed Forces, 2016–2024

**Published:** 2026-01-26

## Abstract

This report summarizes incidence rates and trends of the 5 most frequently occurring sexually transmitted infections (STIs) from 2016 through 2024 among active component service members of the U.S. Armed Forces. The data for this report were derived from medical and public health surveillance of chlamydia, gonorrhea, and syphilis as nationally notifiable diseases; case data for 2 additional STIs, human papillomavirus (HPV) and genital herpes simplex virus (HSV), are also presented. Chlamydia infections were the most common during the surveillance period, followed, in decreasing order of frequency, by HPV, gonorrhea, genital HSV, and syphilis. In 2024, both chlamydia and gonorrhea rates dropped to their lowest points of the period of surveillance, falling 25.5% and 26.4%, respectively, from their 2019 peaks. Declines were predominantly concentrated among service members younger than 25 years of age—who were the largest contributors to overall incidence. Notably, syphilis incidence rose steadily throughout the surveillance period, among all age groups, and both sexes, with steepest rises after 2021, increasing nearly 70%. Non-Hispanic Black service members continue to bear the highest syphilis burden, among whom incidence peaked in 2023, before declining approximately 15% in 2024. Syphilis rates continued to rise among all other racial and ethnic groups through 2024, with the largest relative increase, 456%, among non-Hispanic White service women under age 25 years. Genital HSV demonstrated a downward trend throughout the surveillance period, with overall incidence reaching its lowest point in 2024. Incidence of genital HPV also decreased among all service members, with a more pronounced decrease among men.

**What are the new findings?**

Chlamydia, gonorrhea, and genital HSV incidence rates dropped to their lowest points of the 9-year surveillance period. In contrast, total syphilis incidence rose among all age groups, and both sexes, with the highest incidence among service women ages 17-19 years. While syphilis incidence rates remain highest among non-Hispanic Black service members, its incidence has risen sharply in all other racial and ethnic groups, reflecting an evolving and expanding syphilis epidemiology within the military in addition to the general U.S. population.

**What is the impact on readiness and force health protection?**

STIs can adversely affect service member ability and availability to perform assigned duties and can result in serious medical sequelae if left untreated. Syphilis infection in reproductive age military women can cause miscarriage, stillbirth, or congenital syphilis, affecting women's health, deployability, and overall force readiness, while increasing health care costs. Expanded prevention, testing, and treatment, along with comprehensive sexual health education, particularly targeting those younger than age 25 years, are warranted to curb transmission and maintain operational effectiveness.

Sexually transmitted infections (STIs) represent one of the highest health care burdens attributable to infectious diseases among active component service members (ACSMs) of the U.S. Armed Forces. ^1^ A National Academies of Sciences, Engineering and Medicine committee, convened to provide recommendations for prevention and control of STIs in the U.S., concluded that military recruits and active duty service members warrant focused consideration due to their elevated risk of STIs. ^2^ While multiple and inter-related factors influence STI risk within military populations, the strongest risk factors are age and sex. ^3^ Since the military population consists of young (mean age 26 years) and predominantly male (85%) service members, rates are not directly comparable to the general U.S. population, unless adjusted for those demographics.

The U.S. Centers for Disease Control and Prevention (CDC) publishes annual summaries of national surveillance data for notifiable diseases, including *Chlamydia trachomatis* (chlamydia), *Neisseria gonorrhoeae* (gonorrhea), and *Treponema pallidum* (syphilis), under federally funded control programs. ^4^ Although relatively common bacterial STIs are curable with antibiotics, there is continued concern about the threat of multi-drug resistance. ^5 - 7^

Common viral STIs in the U.S. include infections caused by human papillomavirus (HPV) and genital herpes simplex virus (HSV). ^8 , 9^ While most HPV infections resolve spontaneously, a subset can persist and progress to HPV-associated cancers, including cervical cancer in women, as well as anal, penile, and oropharyngeal cancers in both sexes. ^10^ Similarly, genital HSV can lead to recurrent genital ulcer disease with sustained transmission within the population due to asymptomatic shedding. Suppression of recurrent herpes is attainable using anti-viral medication, and a vaccine prevents infection from 4 of the most common HPV serotypes, as well as 5 additional cancerous types. ^11^

This report presents an update to the previous *MSMR* article on these 5 STIs among U.S. ACSMs, covering the surveillance period of 2016 through 2024. ^12^

## Methods

The surveillance population for this report consists of all ACSMs of the U.S. Army, Navy, Air Force, or Marine Corps who served at any time during the surveillance period of January 1, 2016 through December 31, 2024. Diagnoses of STIs were ascertained from medical administrative data and reports of notifiable medical events routinely provided to the Armed Forces Health Surveillance Division and maintained in the Defense Medical Surveillance System (DMSS) for health surveillance. STI cases were also derived from positive laboratory test results recorded in the Health Level 7 (HL7) chemistry and microbiology databases compiled by the Defense Centers for Public Health–Portsmouth.

The number of days in active service for each service member was ascertained, which were then aggregated to a total for all service members for each calendar year. The resultant annual totals are expressed as person-years (p-yrs) of service, used as the denominators for calculating annual incidence rates. Person-time not considered time at risk for an STI was excluded, such as the 30 days following each incident chlamydia or gonorrhea infection and all person-time following an initial diagnosis, medical event report, or positive laboratory test of HSV, HPV, or syphilis. Incidence rates were calculated as incident cases of a given STI per 100,000 p-yrs of active component service, with percent changes in incidence calculated by un-rounded rates.

An incident case of chlamydia was defined by either 1) a case-defining diagnosis **(Table 1)** in the first or second diagnostic position of a record of an outpatient or intheater medical encounter, 2) a confirmed notifiable disease report, or 3) a positive laboratory test (for any specimen source or test type). An incident case of gonorrhea was similarly defined by 1) a case-defining diagnosis in the first or second diagnostic position of an inpatient, outpatient, or intheater encounter record, 2) a confirmed notifiable disease report, or 3) a positive laboratory test (for any specimen source or test type). For both chlamydia and gonorrhea, an individual could be counted as having a subsequent case only if more than 30 days occurred between the dates recorded for each case-defining diagnosis.

**TABLE 1.** ICD-9 and ICD-10 Diagnostic Codes Used to Identify STI Cases in Electronic Health Care Records

STI	ICD-9	ICD-10
HPV	078.11, 079.4, 795.05, 795.09, 795.15, 795.19, 796.75, 796.79	A63.0, R85.81, R85.82, R87.81, R87.810, R87.811, R87.82, R87.820, R87.821, B97.7
Chlamydia	099.41, 099.5 *	A56. *
Genital HSV	054.1 *	A60. *
Gonorrhea	098. *	A54. *
Syphilis	091. * , 092. * , 093. * –096. * , 097.0, 097.1, 097.9	A51. * (excluding A51.31), A52. * , A53.0, A53.9

Abbreviations: ICD-9, International Classification of Diseases, 9th Revision; ICD-10, International Classification of Diseases, 10th Revision; STI, sexually transmitted infection; HPV, human papillomavirus; HSV, herpes simplex virus.

* Note: Asterisk (*) indicates that any subsequent digit or character is included.

An incident case of syphilis was defined by either 1) a qualifying International Classification of Diseases, 9th or 10th Revision (ICD-9/ICD-10) code in the first, second, or third diagnostic position of a hospitalization record, 2) at least 2 outpatient or in-theater encounters within 30 days with a qualifying ICD-9/ICD-10 code in the first or second position, 3) a confirmed notifiable disease report for any type of syphilis, or 4) a record of a positive polymerase chain reaction or treponemal laboratory test. Stages of syphilis (primary, secondary, late, latent) could not be distinguished because HL7 laboratory data do not allow for stage differentiation, and because a high degree of misclassification is associated with use of ICD diagnosis codes for stage determination. ^13 , 14^ An individual could be considered an incident case of syphilis only once during the surveillance period; those with evidence of prior syphilis infection were excluded.

Incident cases of genital HSV were identified by either 1) presence of requisite ICD-9/ICD-10 codes in either the first or second diagnostic positions of an outpatient or in-theater encounter record or 2) a positive laboratory test from a genital specimen source. Antibody tests were excluded because they do not allow distinction between genital and oral infections. Incident cases of genital HPV were similarly identified by either 1) presence of requisite ICD-9/ICD-10 codes in either the first or second diagnostic positions of an outpatient or intheater encounter record or 2) a positive laboratory test from any specimen source or test type. Outpatient encounters for HPV with evidence of HPV immunization within 7 days before or after an encounter date were excluded, as were outpatient encounters with a procedural or Current Procedural Terminology (CPT) code indicating HPV vaccination, as such encounters were potentially related to vaccination administration. An individual could be counted as an incident case of HSV or HPV only once during the surveillance period. Individuals with diagnoses of HSV or HPV infection before the surveillance period were excluded.

To characterize trends during the surveillance period, age- and sex-specific percent changes relative to each group's peak rate were calculated. Recent trends were assessed through annual percentage changes that compare 2024 with 2023. When notable differences in rates or trends were observed, absolute differences in incidence rates from peak levels were calculated to identify which age- and sex-specific groups contributed most to the overall decline. Results are presented as age- and sex-specific trends for the entire 2016–2024 surveillance period, and as recent changes from 2023 to 2024, for each STI. Incidence rates are expressed per 100,000 p-yrs.

## Results

### General incidence and distribution patterns

Chlamydia infections were the most common during the surveillance period, followed, in decreasing order of infection frequency, by HPV, gonorrhea, genital herpes, and syphilis **(Table 2)** . Chlamydia accounted for the majority of reported STI cases during the surveillance period, with nearly twice as many cases as the combined total of the other 4 STIs, and nearly 5-fold higher than HPV, the next most frequently identified STI. Except for syphilis, incidence was generally higher in female service members; for gonorrhea, total incidence rates were similar between sexes.

**TABLE 2.** Incident Counts and Incidence Rates of STIs, Active Component, U.S. Armed Forces, 2016–2024

		Chlamydia		Gonorrhea		Syphilis		Genital HSV		Genital HPV	
	No.	Rate ^a^	No.	Rate ^a^	No.	Rate ^a^	No.	Rate ^a^	No.	Rate ^a^
Total	227,653	1,955.8	38,987	334.5	7,466	64.2	24,276	211.4	47,629	424.2
Sex										
Male	142,375	1,472.5	30,978	320.1	6,412	66.4	12,848	134.1	15,099	158.8
Female	5,278	4,327.4	8,009	405.1	1,054	53.4	11,428	601.2	32,530	1,893.3
Age group, y										
<20	31,654	3,810.0	4,154	498.7	803	96.4	1,811	217.6	566	67.9
20–24	132,048	3,585.1	20,235	548.0	2,817	76.4	10,272	280.0	17,038	465.7
25–29	44,083	1,627.7	8,829	325.6	1,910	70.6	6,377	238.4	12,659	481.0
30–34	13,250	706.1	3,584	190.9	1,075	57.4	3,174	172.7	10,405	591.8
35–39	4,797	344.2	1,492	107.0	517	37.2	1,651	122.1	4,319	339.0
>40	1,821	158.7	693	60.4	344	30.1	991	88.9	2,642	246.4
Race and ethnicity										
White, non-Hispanic	82,856	1,303.7	9,910	155.8	2,289	36.0	10,255	163.2	21,431	348.3
Black, non-Hispanic	75,330	4,019.9	19,692	1,048.3	2,648	141.5	7,332	401.1	10,723	600.3
Hispanic	46,940	2,327.5	5,926	293.4	1,675	83.1	4,406	221.1	9,163	470.4
Other, unknown	22,527	1,616.1	3,459	247.9	854	61.3	2,283	165.7	6,312	471.1
Education										
High school or less	197,278	2,683.9	32,457	440.8	5,498	74.8	17,224	236.4	28,265	392.2
Some college	13,538	983.7	2,738	198.8	736	53.6	2,742	204.9	6,482	509.1
Bachelor's, advanced degree	14,331	543.9	3,317	125.8	1,088	41.4	3,892	150.7	11,579	466.9
Other, unknown	2,506	900.3	475	170.6	144	51.8	418	152.0	1,303	484.4
Marital status										
Single, never married	162,191	3,157.3	26,853	521.6	4,842	94.2	13,292	260.3	23,341	462.2
Married	51,802	873.3	9,874	166.4	2,170	36.6	8,516	146.1	18,648	328.9
Other, unknown	13,660	2,390.8	2,260	394.9	454	79.6	2,468	451.6	5,640	1,111.9
Service branch										
Army	93,258	2,234.6	18,706	447.6	2,753	66.0	10,224	248.6	16,474	407.8
Navy	54,587	1,841.9	9,662	325.6	2,790	94.3	5,977	204.5	13,991	491.0
Air Force	44,841	1,549.4	5,971	206.1	1,233	42.6	5,454	191.4	12,782	463.8
Marine Corps	34,967	2,173.6	4,648	288.5	690	42.9	2,621	164.1	4,382	276.8
Rank, grade										
Junior enlisted (E1–E4)	169,188	3,415.8	26,816	540.1	4,786	96.6	13,227	267.9	21,825	443.9
Senior enlisted (E5–E9)	49,083	1,066.8	10,144	220.3	2,092	45.6	8,307	184.9	17,440	403.1
Junior officer (O1–O3)	7,957	681.1	1,522	130.2	401	34.4	1,979	171.3	6,083	539.7
Senior officer (O4–O10)	848	113.3	348	46.5	138	18.5	554	75.6	1,833	261.9
Warrant officer (W01–W05)	577	341.2	157	92.8	49	29.0	209	127.8	448	285.1
Military occupation										
Combat-specific ^b^	26,703	1,689.0	4,783	302.2	670	42.4	2,501	159.5	3,298	212.2
Motor transport	10,970	3,217.9	2,087	610.9	431	126.5	920	272.8	1,816	546.2
Pilot, air crew	2,273	543.3	315	75.3	92	22.0	454	109.9	1,162	288.3
Repair, engineering	62,948	1,851.1	10,486	308.0	1,667	49.1	6,204	184.5	11,147	336.8
Communications, intelligence	56,561	2,268.1	10,751	430.5	1,658	66.6	6,611	270.6	13,396	567.7
Health care	17,014	1,703.9	2,897	289.8	619	62.1	2,637	269.5	7,196	771.9
Other	51,184	2,126.8	7,668	318.2	2,329	96.8	4,949	208.0	9,614	411.5

Abbreviations: STIs, sexually transmitted infections; HSV, herpes simplex virus; HPV, human papillomavirus; No., number; y, years.

a Incidence rate per 100,000 person-years.

b Infantry, artillery, combat engineering, armor.

The highest concentration of cases was among service members ages 20-24 years, who comprised over half of chlamydia (58%) and gonorrhea (52%) cases, as well as 36-42% of cases for the other 3 STIs. The 25-29-years age group accounted for the next largest proportion of cases for all STIs, comprising 19-27% of cases. HPV was most common (22%) STI among those aged 30-34 years.

Incidence rates for all 5 STIs were highest among those who had never married (among those with defined marital status) as well as non-Hispanic Black service members. With the exception of HPV, rates of infection were also highest among individuals with a high school education or less, and among junior enlisted members; in contrast, HPV revealed a different pattern, with the highest incidence observed among those with educations beyond high school, and among junior officers. Chlamydia and gonorrhea incidence were highest in the Army, syphilis and genital HPV incidence were highest in the Navy, while genital HSV rates were generally comparable among service branches.

### Chlamydia

### Age- and sex-stratified trends

**FIGURE 1.**
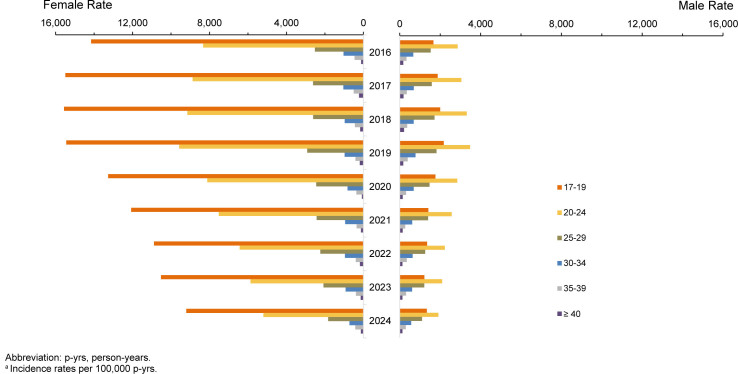
Incidence Rates ^a^ of *Chlamydia Trachomatis* Infection Among Women and Men, by Age, Active Component, U.S. Armed Forces, 2016–2024

Annual chlamydia rates continued to decline in 2024, extending the downward trend observed since 2020, as previously reported **(Figure 1)** . ^12^ In 2024, chlamydia incidence fell to its lowest point in 9 years, to 1,395.9 cases per 100,000 p-yrs, a 43.8% decline from the 2019 peak rate of 2,484.1 cases per 100,000 p-yrs. The largest reductions from peak rates occurred in younger age groups, who accounted for most of the total incidence rate decline. Among female service members, over 88% of the total decline (12,144 fewer overall cases per 100,000 p-yrs; 46.5% decrease from 2019 peak) was among women ages 17-24 years (data not shown). Among male service members, the largest declines were among those aged 20-24 years (1,570 fewer cases per 100,000 p-yrs; 45.1% decrease from peak), followed by those aged 17-19 years (838 fewer cases per 100,000 p-yrs, 38.5% decrease), and 25-29 years of age (716 fewer cases per 100,000 p-yrs, 39.3% decrease) (data not shown).

Chlamydia rates among female service members were generally 3 times higher than among male service members throughout the 9-year surveillance period. Throughout the surveillance period, for individuals aged 17-19 years, rates were 7–9 times higher among women than men. Older groups (>age 30 years) accounted for a much smaller share of the total incidence and contributed minimally to overall declines in both sexes. Declines in chlamydia rates were consistent among all racial and ethnic groups **(Figure 2)** .

**FIGURE 2a.**
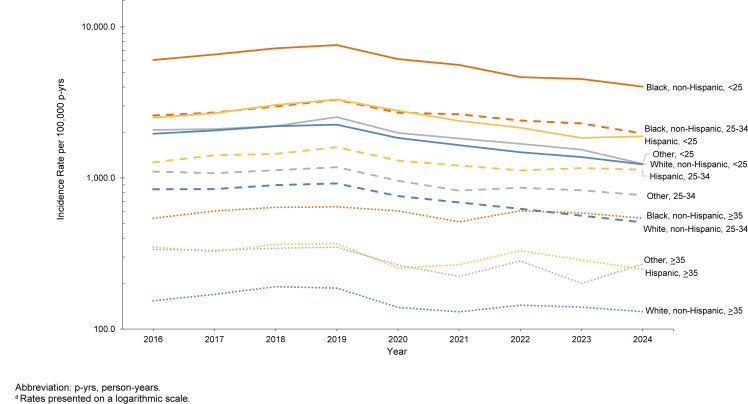
Incidence Rates ^a^ of *Chlamydia Trachomatis* Infection Among Women, by Age and Racial and Ethnic Group, Active Component, U.S. Armed Forces, 2016-2024

**FIGURE 2b.**
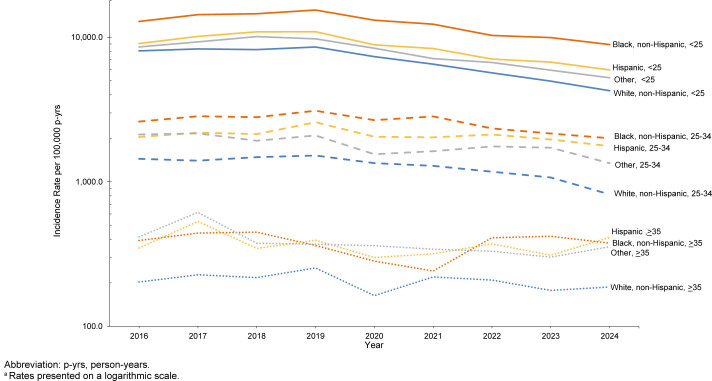
Incidence Rates ^a^ of *Chlamydia Trachomatis* Infection Among Men, by Age and Racial and Ethnic Group, Active Component, U.S. Armed Forces, 2016-2024

### Age- and sex-specific changes in 2024 versus 2023

Total chlamydia incidence rates declined by 11.8% (from 3,310.4 cases per 100,000 p-yrs in 2023 to 2,919.5 cases per 100,000 p-yrs in 2024) among female and 7.9% (from 1,155.6 cases per 100,000 p-yrs in 2023 to 1,064.7 cases per 100,000 p-yrs in 2024) among male service members. Declines among female service members were concentrated among those aged 17-24 years, with 17-19-year-olds driving the largest decrease: 12.5% (data not shown). In contrast, rates among male service members in the same 17-19-years age range increased by 9.7%. Divergent changes were also observed among older age groups (35-39 years), with incidence rates increasing (+10.2%) among women and decreasing (-4.4%) among men. In the 40 years and older age group, differences were minor, with slight declines in women (-5.5%) and small increases (+2.2%) in men.

### Gonorrhea

### Age- and sex-stratified trends

**FIGURE 3.**
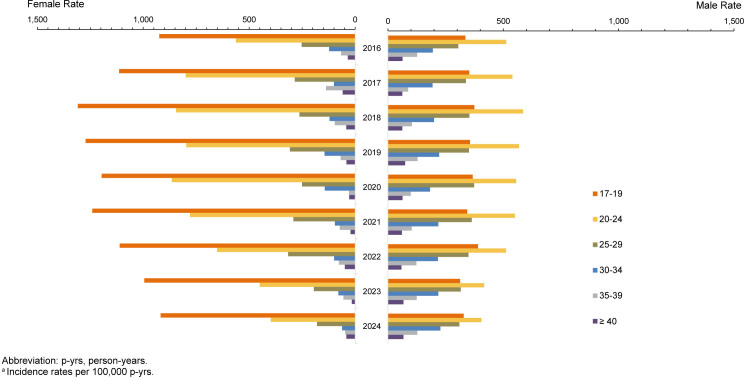
Incidence Rates ^a^ of Gonorrhea Infection Among Women and Men, by Age, Active Component, U.S. Armed Forces, 2016–2024

Gonorrhea incidence rates continued to decline for both female and male service members in 2024, following increases that peaked prior to 2020. These trends parallel those observed for chlamydia **(Figure 3)** . The largest reductions in gonorrhea incidence occurred among younger age groups. Among female service members, total crude incidence decreased from 490.9 per 100,000 p-yrs at the 2018 peak to 254.9 per 100,000 p-yrs in 2024 (-48.1%), with those younger than age 25 years accounting for 72.4% of this reduction. Similarly, the total crude incidence among male service members declined from 347.0 per 100,000 p-yrs at the 2019 peak to 276.6 per 100,000 p-yrs in 2024 (-20.3%), with those younger than age 30 years accounting for nearly all (98.8%) of the total decline. Women older than age 25 years also experienced notable declines (range -42% to -66%), while incidence among men aged 30 years and older remained relatively stable over the surveillance period.

As observed with chlamydia infections, female service members aged 17-19 years demonstrated highest gonorrhea incidence, with rates nearly 3 times higher than their male counterparts. Sex disparities in other age groups were less pronounced, although men older than age 35 years tended to have higher gonorrhea rates than women of the same age (data not shown).

### Age- and sex-specific changes in 2024 versus 2023

Gonorrhea incidence among service women declined by 9.3% from 2023 to 2024, with the largest decrease among those aged 30-34 years (-22.9%). The total rate among service men declined slightly (-1.2%), with no notable changes in age-stratified rates (data not shown).

### Syphilis

### Age- and sex-stratified trends

**FIGURE 4.**
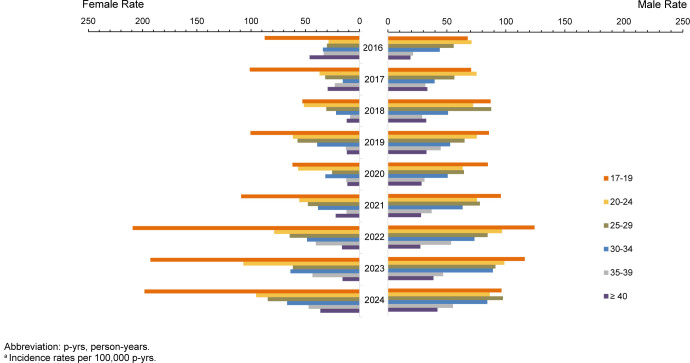
Incidence Rates ^a^ of Syphilis Infection Among Women and Men, by Age, Active Component, U.S. Armed Forces, 2016–2024

**FIGURE 5.**
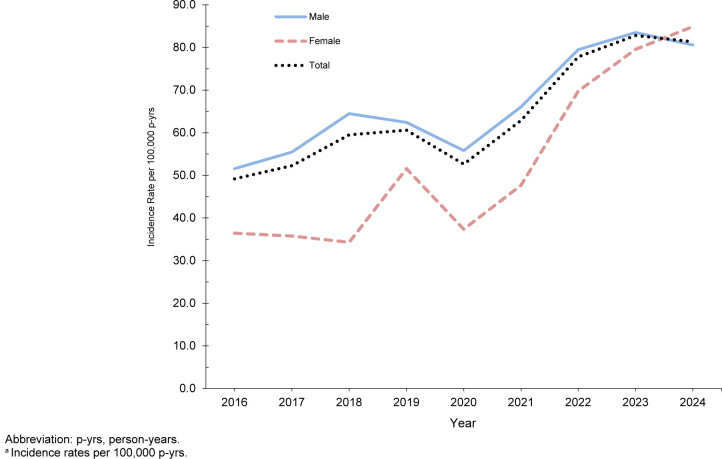
Incidence Rates ^a^ of Syphilis by Sex, Active Component, U.S. Armed Forces, 2016–2024

Syphilis incidence increased from 2016 through 2024 among all age groups, and both sexes, with the largest increases observed after 2021 **(Figure 4)** . In 2024 a remarkable shift occurred in syphilis incidence: The total incidence rate among female service members surpassed that of male service members for the first time during the surveillance period **(Figure 5)** . The steepest increase among service women was observed in those aged 17-19 years, among whom incidence rose nearly 4-fold, from a low of 52.7 per 100,000 p-yrs in 2018 to approximately 200 cases per 100,000 p-yrs from 2022 through 2024. Rates among women rose approximately 3-fold from 2016 to 2024 among those aged 20-24 years, from 28.7 cases per 100,000 p-yrs in 2016 to 95.4 cases per 100,000 p-yrs in 2024, with a peak of 107.1 cases in 2023. Although incidence rates were lower in the older female age groups, they demonstrated 3-to 5-fold increases.

Syphilis incidence among service men aged 17-19 years peaked at 124.4 per 100,000 p-yrs in 2022, before declining to 96.4 per 100,000 p-yrs in 2024, which still represented a 1.4-fold increase from the 2016 low of 67.7 per 100,000 p-yrs. The 20-24-years male age group showed a smaller (1.2-fold) increase from 2016 to 2024, while incidence rates among men ages 25-34 years increased 1.7- to 1.9-fold, reaching incidence rate levels comparable to the youngest age groups in 2024. Substantial rises in incidence were also observed among older males: 2.6-fold (from 21.5 to 55.2 per 100,000 p-yrs) among those aged 35-39 years and 2.2-fold (from 19.1 to 42.1 per 100,000 p-yrs) among those aged 40 years and older.

Syphilis burden was highest among non-Hispanic Black service members, with incidence rates 2- to 5-times greater (depending upon age group) than those of non-Hispanic White service members, who had the lowest incidence. Non-Hispanic Black men younger than age 25 years accounted for a disproportionate number of syphilis cases, and had the highest incidence rates, which peaked in 2023 at 271.9 per 100,000 p-yrs before declining to 197.1 (-27.5%) per 100,000 p-yrs in 2024. Rates among non-Hispanic Black women younger than age 25 years peaked at 196.9 per 100,000 p-yrs in 2023, followed by 15.1% decline to 167.1 per 100,000 p-yrs in 2024.

Despite lower baseline levels, other racial and ethnic groups demonstrated substantial increases in syphilis incidence throughout the surveillance period. In particular, among women younger than age 25 years, the largest relative increase, 455.5%, was observed among non-Hispanic White women, whose rate in 2024 was the highest (11.3 per 100,000 p-yrs in 2016 to 62.9 per 100,000 p-yrs in 2024), followed by service members in the ‘other’ (+398.1%, from 27.7 in 2017 to 137.8 in 2024) and Hispanic racial and ethnic categories (+303.4%, from 42.4 in 2016 to 171.0 in 2023).

In general, female service members had lower syphilis rates than their male counterparts, but among those aged 17-19 years, female rates exceeded male rates during 7 of the 9 years of surveillance. Rise in overall incidence of syphilis among service men was relatively modest, with the smallest increase (+27.6%) observed among Hispanic service members, and the largest (+68.2%) among non-Hispanic Black service members.

### Age- and sex-specific changes in 2024 versus 2023

Changes in syphilis rates in 2024 compared to 2023 diverged by sex. Overall incidence increased from 79.5 per 100,000 p-yrs in 2023 to 84.9 per 100,000 p-yrs in 2024 (+6.7%) among women but declined 3.5% among men (from 83.5 to 80.6 per 100,000 p-yrs). Incidence among women increased among all age groups, except those aged 20-24 years, among whom syphilis declined by 11%, from 107.1 in 2023 to 95.4 per 100,000 p-yrs in 2024. Decreases among men were concentrated in younger age groups, particularly those aged 17-24 years (-16.9%, from 116.0 in 2016 to 96.4 in 2024). In contrast, incidence increased among older men, most notably those aged 35-39 years (+17.9%, from 46.8 in 2016 to 55.2 in 2024).

### Genital human papillomavirus

### Age- and sex-stratified trends

**FIGURE 6.**
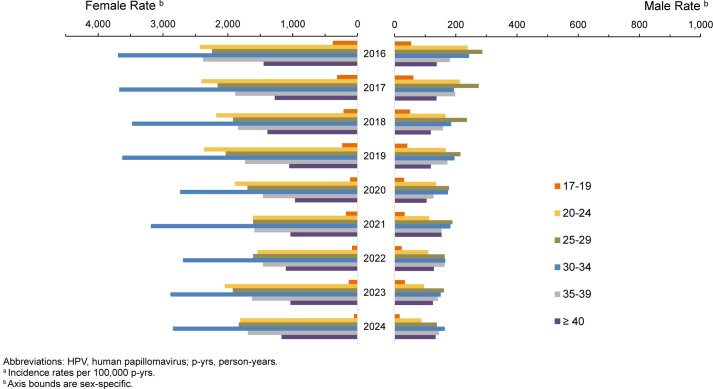
Incidence Rates ^a^ of Genital HPV Infection Among Women and Men, by Age, Active Component, U.S. Armed Forces, 2016–2024

Crude annual incidence rates of genital HPV infections among all ACSMs decreased by 24.1%, from 511.3 cases per 100,000 p-yrs in 2016 to 388.0 cases per 100,000 p-yrs in 2024, with a more pronounced decrease among service men. On average, HPV rates in female service members were 10 times higher than those of male service members. Incidence rates of genital HPV infections among male service members overall followed a steadily downward trajectory, with a minor uptick in 2021, decreasing from a high of 220.6 cases per 100,000 p-yrs in 2016 to the lowest level, 119.7 cases per 100,000 p-yrs, in 2024 (-45.7%) **(Figure 6)** . Incidence among female service members declined from a high of 2,278.8 per 100,000 p-yrs in 2016 to 1,775.7 cases per 100,000 p-yrs in 2024 (-22.1%), with the lowest point in 2022, at 1,584.4 cases.

**FIGURE 7.**
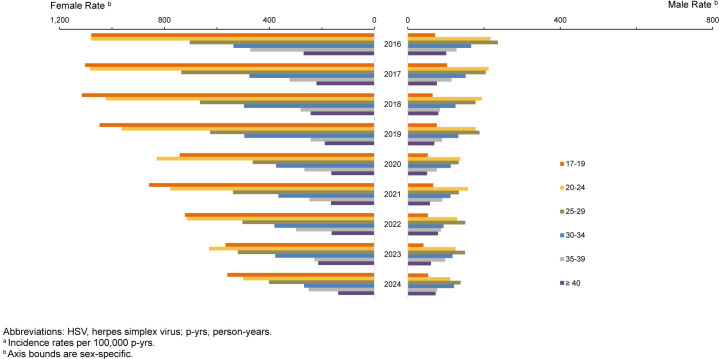
Incidence Rates ^a^ of Genital HSV Infection Among Women and Men by Age Group, Active Component, U.S. Armed Forces, 2016–2024

Service women in the 17-19-years age group showed the largest reduction (-85.8%) in genital HPV, dropping from 381.3 per 100,000 p-yrs in 2016 to 54.0 per 100,000 p-yrs in 2024. Declines in older age groups were modest, ranging from approximately 19% to 29%. Among those aged 30-34 years—the female age group with the largest detection rate of HPV—incidence decreased by 23% from 3,694.2 to 2,845.4 cases per 100,000 p-yrs from 2016 to 2024.

Declines among service men were pronounced from their peak levels for most age groups. The magnitude of reduction progressively decreased with increasing age, with the greatest drop observed among those aged 17-19 years (-83.0%, from 61.7 in 2017 to 16.8 per 100,000 p-yrs in 2024), followed by those aged 20-24 years (-63.2%, from 238.9 in 2016 to 88.0 in 2024), and 25-29 years (-51.6%, from 286.4 in 2016 to 138.5 in 2024). Older groups of male service members experienced more gradual declines, with those aged 30-39 years experiencing an approximately 30% decrease over the entire surveillance period, and those aged 40 years and older group remaining largely stable, declining by only 2.8% from 137.7 in 2016 to 133.9 in 2024.

### Age- and sex-specific changes in 2024 versus 2023

The magnitude of annual reduction in HPV incidence among service women from 2023 to 2024 progressively decreased with increasing age, from 59.9% (134.8 in 2023 to 54.0 per 100,000 in 2024) among those aged 17-19 years, to 1.4% (2,886.2 in 2023 to 2,845.4 per 100,000 p-yrs in 2024) among those aged 30-34 years. In older female age groups, this trend reversed, with rates among those aged 35-39 years increasing 4.1% (from 1,625.3 in 2023 to 1,691.3 per 100,000 p-yrs in 2024), and among those aged 40 years and older, rates increased 13.1% (from 1,035.4 in 2023 to 1,171.5 per 100,000 p-yrs in 2024). Among men, the youngest (17-19-years) age group continued to decline sharply, from 33.6 in 2023 to 16.8 in 2024 (-49.9%), with moderate levels of decline, 9–14%, among men aged 20-29 years. Similar to the HPV rate declines among women, incidence rates among men older than age 30 years showed a reversal of trending declines, rebounding 3-9%, which indicates a shift in the HPV burden towards older ages in both sexes.

### Genital herpes simplex virus

### Age- and sex-stratified trends

From 2016 through 2024, both female and male service members experienced substantial declines in HSV, but their extents and patterns differed. Total female incidence fell from 773.9 cases per 100,000 p-yrs in 2016 to 382.9 cases per 100,000 p-yrs in 2024 (-50.5%). Male incidence decreased from 180.9 in 2016 to 107.4 in 2024 (-40.6%). The largest declines for both male and female service members occurred among those aged 20-24 years (-53.8% and -48.6%, respectively). Other age groups also experienced notable decreases during the surveillance period, more pronounced among females. Women aged 17-19 years had approximately 3 times as many cases as their male counterparts, with the incidence rate per 100,000 revealing a female-to-male rate ratio of about 14:1. The rate ratio declined with increasing age.

### Age- and sex-specific changes in 2024 versus 2023

In 2024, changes in patterns diverged by sex. Total incidence rates among women fell nearly 20%, from 478.0 in 2023 to 382.9 per 100,000 p-yrs in 2024, among all age groups except those aged 35-39 years, among whom HSV increased by 9.6%, from 228.5 to 250.3 per 100,000 p-yrs. In contrast, male trends indicated more variable results, with total rates declining by only 6% (from 114.4 to 107.4 per 100,000 p-yrs) while increasing by over 20% among individuals aged 17-19 years (from 41.2 to 53.5 per 100,000) and over age 40 years (from 60.9 to 73.2 per 100,000) (data not shown).

**FIGURE 8.**
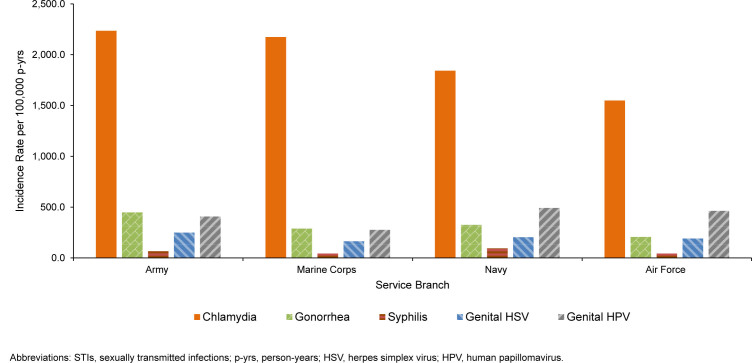
Incidence Rates of STIs, Active Component, U.S. Armed Forces, 2016–2024

## Discussion

This report provides a surveillance update on 3 nationally notifiable bacterial STIs—chlamydia, gonorrhea, and syphilis—as well as 2 viral STIs, genital HSV and HPV. Chlamydia was the most frequently reported STI during the surveillance period, with total cases and incidence rates exceeding those of HPV, the secondmost common STI, approximately 5 times. Gonorrhea was the third most reported STI, followed by genital HSV and syphilis.

### Chlamydia and gonorrhea

Chlamydia and gonorrhea are both bacterial infections that are frequently asymptomatic and typically tested together due to shared screening programs and diagnostic laboratory methods. ^15^ Consequently, temporal trends in incidence rates for both infections tend to reflect the other, as observed in this analysis.

During the initial 4 years of the surveillance period, both chlamydia and gonorrhea showed upward trends, peaking in 2019, before declining in 2020. The declines for the 2 STIs persisted through 2024: 40% for both infections among service women, and over 20% for gonorrhea among service men. Women under age 25 years and men under age 30 years accounted for most cases, who, correspondingly, experienced the largest reductions in incidence.

The trend pattern observed over the past 5 years aligns with the recent CDC data, which indicate that between 2019 and 2023, the total rates of both chlamydia and gonorrhea decreased among general population women by approximately 12–14% and among men by approximately 8%. ^16^ Gonorrhea, however, showed little change, or increased slightly, by approximately 2%, among men in the general population. Over the longer period, from 2014 through 2023, CDC data show that chlamydia rates in the general population diverged between sexes, increasing by 33.4% among men and decreasing by only 1.8% among women.

In contrast, in this analysis, which covers a comparable period, from 2016 through 2024, chlamydia rates among ACSMs demonstrated relatively steady downward trend, declining by more than 30% in both sexes. Corresponding rates for gonorrhea showed decreases by 10.1% among service men and 26.6% among service women. Chlamydia incidence rates were markedly higher among service members, with rates among males approximately 4–5 times, and females about 7 times, higher than those of civilian counterparts. Corresponding military to civilian ratios for gonorrhea were 1.4–2.7 times higher among men and 3.1–4.0 times higher among women.

Higher rates in military populations are likely due to a combination of demographic, behavioral, and structural factors. The military population is predominantly young, highly mobile, with frequent relocations and deployments, and often residing in close social environments, which are factors known to increase the risk of STI acquisition. ^2 , 13^ Additionally, the military implements aggressive screening programs (e.g., routine and mandatory testing among women younger than age 25 years) to maintain a fit and ready military force, coupled with no-cost access to preventive and primary care, which facilitate more comprehensive case detection. ^17 , 18^ Electronic health records within the Military Health System (MHS) further enable more complete disease burden capture for notifiable disease reporting. Nevertheless, these rate comparisons should be interpreted cautiously, as differences in surveillance and reporting practices between military and civilian populations may introduce surveillance bias. ^19^

Laboratory and medical encounter data from service members in 2022 supplemented chlamydia case rates, as those cases had no medical event report and would have been unidentifiable with-out supplemental electronic health record data. Routine surveillance reports do not assess anatomical sites from gonorrhea case reports and laboratory records, which could provide more comprehensive understanding of extragenital infections in high risk populations.

National guidelines recommend gonorrhea screening, including pharyngeal or rectal testing, at least annually for both men who have sex with men (MSM) and HIV-positive patients. Extragenital gonorrhea screening may be considered for women on the basis of reported sexual behaviors and exposure. ^20^ Despite these recommendations, extragenital screening for high risk civilian and military populations is under-used. ^21 , 22^ A recent assessment of extragenital STI screening by primary care physicians for HIV-positive male Air Force service members found that approximately one-third of patients had undetected STIs, the majority due to extragenital infections of the rectum and pharynx. ^22^

### Syphilis

The trend in syphilis rates reveals a pattern that differs from the other 2 bacterial STIs, reflecting differing epidemiological factors and clinical dynamics. Total syphilis incidence trends mirror national trends in the civilian population. CDC data indicate that rates of primary and secondary syphilis among women in the general U.S. population increased nearly 5-fold nationally from 2015 through 2024, rising 392.9% from 1.4 to 6.9 cases per 100,000 population. In contrast, corresponding rates among men over the same period increased 29.4%, from 13.6 to 17.6 cases per 100,000 population. Syphilis rates were highest among non-Hispanic Black service members of both sexes, consistent with national data evidencing persistently elevated rates in this population. While syphilis incidence among non-Hispanic Black service members declined in 2024, they continue to bear the highest burden of infection, evincing the persistence of syphilis in this population and potentially reflecting ongoing challenges in delivering effective prevention, testing, and treatment services.

Conversely, syphilis incidence among service members of other racial and ethnic groups, especially women younger than age 25 years, continued to increase through the end of the surveillance period. The largest relative increase was observed among non-Hispanic White female service members, a group previously associated with the lowest syphilis burden, increasing 456% from 2016 to 2024. These trends correlate with the findings of a recent study that found declining syphilis incidence among historically highly-burdened population groups, while concurrently increasing in lesser-burdened groups. ^23^

The sharp rise in syphilis incidence observed among reproductive age women is a significant public health concern due to the risk of maternal and congenital syphilis. The rate of maternal syphilis among female MHS beneficiaries rose by 233% from 2012 to 2022, while the rate of congenital syphilis among newborn MHS beneficiaries increased by 355%. ^24^ Nationally, the maternal syphilis rate increased by 222% from 2016 to 2022, and congenital syphilis cases rose 700% over the past decade, from 2015 to 2024. ^25 , 16^ These findings indicate critical gaps in prevention, screening and treatment for young service women, and reinforce U.S. Preventive Services Task Force recommendations for early syphilis infection screening in all pregnant women. ^26^

This cycle of syphilis resurgence appears to have begun in the early 2000s, in both civilian and military populations, with steady and notable increases among active component service members reported since the early 2010s. ^27 , 28^ Early increases were primarily attributed to MSM, a group also at elevated risk for HIV infection. Recent data suggest that among MSM, especially those under age 25 years, syphilis and HIV infections may increasingly co-occur, underscoring the need for integrated prevention and control strategies that address both infections concurrently. ^16 , 29^

Collectively, these findings suggest shifting syphilis epidemiology, from the moderate resurgence in 2010s described by Garges in 2016 ^28^ to a sustained and broader increase across the force. Further studies are needed to understand the underlying drivers of these trends, including sexual behaviors and risk factors that are influenced by military service, the reach and effectiveness of existing prevention and screening programs, and unique “social context of soldiers, sailors, airmen, and marines” ^28^ affecting syphilis transmission.

### Human papillomavirus

HPV rates among male and female service members declined steadily over the surveillance period, with the largest reductions, over 80%, observed in the youngest cohorts (ages 17-19 years) of both sexes. These data are consistent with CDC data that show the incidence of HPV infection, particularly in younger populations, declining significantly since the introduction of the HPV vaccine in 2006. ^30^ Specifically, the prevalence of vaccine type HPV strains (6, 11, 16, 18) dropped 86% among young women ages 14-19 years within the decade following vaccine introduction. ^31^

Vaccination alone, however, does not fully explain the drastic decline observed among the youngest service members, who now represent almost a negligible proportion of the total recorded burden of HPV. Previous studies have documented suboptimal vaccine uptake among service members. ^32 , 33^ Between 2007 and 2017, only approximately 27% of eligible service women and 6% of service men initiated HPV vaccination, and completion of the 3-dose series was even lower. ^33^ Contributing factors include inadequate awareness and education, lack of centralized vaccine monitoring within the MHS, the voluntary nature of vaccination, and the mobile life style of service members. ^34^ It is also possible that HPV vaccination records are incomplete. The HPV vaccine is typically recommended during early adolescence (i.e., before military service), and prior vaccination may not have been reported or recorded, leading to an under-estimation of actual vaccination coverage

An additional factor contributing to the dramatic decline in rates of HPV detection among women ages 17-20 years is MHS implementation of updated national cervical cancer screening guidelines, which recommend delaying screening until age 21 years. Furthermore, routine screening is not recommended for men, resulting in under-detection within this population. ^15^

In contrast, HPV detection rates were highest among women ages 20-39 years, with the greatest burden observed in those aged 30-34 years. A strong cohort effect was evident, with the magnitude of HPV rate reductions progressively diminishing with increasing age, and even reversing in 2024 among individuals older than age 35 years. This observed pattern likely reflects lower vaccination among older cohorts, HPV infection persistence, and expanded or more frequent screening practices in those age groups. Supporting this hypothesis, a recent study on cervical cancer screening modalities found that MHS screening practices align with national guideline updates, including increased use of HPV co-testing and expanded screening among women ages 30-64 years. ^34^ Concurrently, cervical cancer screening has decreased among women younger than age 21 years, consistent with recommendations to delay initiation of screening in that age group. ^35^

Targeted efforts are warranted to improve HPV vaccine awareness, accessibility, and completion among service members, and reinforcing education for both health care providers and personnel could strengthen vaccine uptake and help reduce the long-term burden of HPV-related diseases within the armed forces.

### Herpes simplex virus

The trends in the incidence of genital HSV in the U.S. military are consistent with the CDC's National Health and Nutrition Examination Survey (NHANES) rounds that show declining seroprevalence in the U.S. population since the late 1990s. National seroprevalence among individuals aged 14-49 years dropped from 18% in 1999-2000 to around 12% by 2015-2020. ^36 , 37^ Total incidence among service members decreased by roughly 40% to 50%, depending on the age group and sex, between 2016 and 2024.

No sexual risk behavior data were available for this report, but prior surveys of military personnel indicate increased behaviors of possible concern. The 2018 Department of Defense Health Related Behaviors Survey (HRBS) documented that 19.3% of active component respondents reported 2 or more sexual partners within the past year, with 34.9% reporting sex without condom use with a new partner in the past year—percentages almost double those in the 2011 survey. ^38^

This report has several limitations. Changes in incidence rates may reflect, at least in part, temporal changes in case detection, including more aggressive screening. Furthermore, STI diagnoses can be incorrectly coded. For example, STI-specific ‘rule out’ diagnoses or vaccinations (e.g., HPV vaccination) may be reported with STI-specific diagnostic codes, which would result in over-estimated STI incidence.

Cases of syphilis, genital HSV, and genital HPV infections based solely upon laboratory test results are considered ‘suspect’ because laboratory results cannot distinguish between active and chronic infections. Because incident cases of syphilis, genital HSV, and genital HPV were identified based upon a first qualifying encounter or laboratory result, it is likely most cases were acute and not chronic.

STI cases coded in the medical record using symptom codes (e.g., urethritis) rather than STI-specific codes may not be captured. In addition, counts of STI diagnoses reported herein may under-estimate actual diagnoses because some service members may have been diagnosed and treated by non-military health care providers (e.g., county health departments, family planning centers) that were not reimbursed, or in deployed settings (e.g., overseas training exercises, combat operations, aboard ships). Laboratory tests ordered from purchased care or in a shipboard facility, battalion aid station, or in-theater facility were not captured in this analysis.

Lack of standard service and installation practices for STI screening, testing, treatment, and reporting complicates interpretations of detected differences between services, military and demographic subgroups, as well as locations. For some STIs, detection of prevalent infection may occur long after initial infection. Standard STI screening, testing, treatment, and reporting among the services, along with consistent adherence, can improve detection and characterization of STI-related health threats. Continued behavioral risk reduction interventions are still required to counter STIs among military service members.
